# Inhibition of UCH-L1 Deubiquitinating Activity with Two Forms of LDN-57444 Has Anti-Invasive Effects in Metastatic Carcinoma Cells

**DOI:** 10.3390/ijms20153733

**Published:** 2019-07-31

**Authors:** Eiji Kobayashi, Duhyeong Hwang, Anjali Bheda-Malge, Christopher B. Whitehurst, Alexander V. Kabanov, Satoru Kondo, Mitsuharu Aga, Tomokazu Yoshizaki, Joseph S. Pagano, Marina Sokolsky, Julia Shakelford

**Affiliations:** 1 Lineberger Comprehensive Cancer Center, UNC at Chapel Hill, Department of Immunology and Microbiology, University of North Carolina at Chapel Hill, Chapel Hill, NC 27599, USA; 2 Center for Nanotechnology in Drug Delivery and Division of Molecular Pharmaceutics, Eshelman School of Pharmacy, University of North Carolina at Chapel Hill, Chapel Hill, NC 27599, USA; 3 Laboratory of Chemical Design of Bionanomaterials, Faculty of Chemistry, M.V. Lomonosov Moscow State University, 119992 Moscow, Russia; 4 Division of Otolaryngology–Head and Neck Surgery, Graduate School of Medicine, Kanazawa University, Kanazawa 920-8640, Japan

**Keywords:** de-ubiquitination, markers of invasion and metastasis, poly (2-oxazoline) micelle, nanoformulation

## Abstract

Normally ubiquitin C-terminal hydrolase L1 (UCH-L1) is expressed in the central nervous and reproductive systems of adults, but its de novo expression has been detected in many human cancers. There is a growing body of evidence that UCH-L1 de-ubiquitinating (DUB) activity plays a major pro-metastatic role in certain carcinomas. Here we tested anti-metastatic effects of the small-molecule inhibitor of UCH-L1 DUB activity, LDN-57444, in cell lines from advanced oral squamous cell carcinoma (OSCC) as well as invasive nasopharyngeal (NP) cell lines expressing the major pro-metastatic gene product of Epstein–Barr virus (EBV) tumor virus, LMP1. To overcome the limited aqueous solubility of LDN-57444 we developed a nanoparticle formulation of LDN-57444 by incorporation of the compound in polyoxazoline micellear nanoparticles (LDN-POx). LDN-POx nanoparticles were equal in effects as the native compound in vitro. Our results demonstrate that inhibition of UCH-L1 DUB activity with LDN or LDN-POx inhibits secretion of exosomes and reduces levels of the pro-metastatic factor in exosomal fractions. Both forms of UCH-L1 DUB inhibitor suppress motility of metastatic squamous carcinoma cells as well as nasopharyngeal cells expressing EBV pro-metastatic Latent membrane protein 1 (LMP1) in physiological assays. Moreover, treatment with LDN and LDN-POx resulted in reduced levels of pro-metastatic markers, a decrease of carcinoma cell adhesion, as well as inhibition of extra-cellular vesicle (ECV)-mediated transfer of viral invasive factor LMP1. We suggest that soluble inhibitors of UCH-L1 such as LDN-POx offer potential forms of treatment for invasive carcinomas including EBV-positive malignancies.

## 1. Introduction

In the past several years, it has become clear that ubiquitin carboxyl-terminal hydrolase L1 (UCH-L1) is an evolutionarily conserved multifunctional protein which is likely to contribute to a number of cellular physiological activities in normal and transformed cells. Although the cellular functions of this molecule still remain unclear, a number of studies have shown that UCH-L1 participates in nuclear/cytosol and membrane trafficking, regulation of cytoskeleton dynamics, stabilization and activation of signaling molecules, regulation of the pool of free ubiquitin as well as lysosome/proteasome activities [1,2].

Although de novo expression of UCH-L1 has been observed in different types of malignancies [3,4], the function of UCH-L1 in the development of primary tumors remains unclear [3,5,6]: Overexpression of UCH-L1 in in vivo transgenic mouse models induces lymphomas acting as an oncogene [7,8], while in the cell lines and tissue samples from different primary carcinomas the expression of uch-l1 is frequently silenced by promoter methylation, suggesting its potential role as a tumor suppressor [8,9]. Several cellular targets (direct or indirect) have been identified for UCH-L1 in different types of malignancies: UCH-L1 contributes to p27 (Kip1) degradation via its interaction and nuclear translocation with JAB1 (COPS5) in lung cancer cells [10]; the β-catenin oncogenic pathway is activated by UCH-L1 [11]; UCH-L1 manipulates the mTOR-mediated protein biosynthesis and is required for MYC-driven lymphomagenesis in mice [12]. Tumor viruses such as the Epstein-Barr virus (EBV), human papillomavirus (HPV), and Kaposi’s sarcoma-associated herpesvirus (KSHV) also induce *UCH-L1* expression during cell transformation [13,14,15,16,17]. Despite some controversy on the functional role of UCH-L1 in the development of primary tumors, the ability of UCH-L1 to promote malignant progression, namely invasion and metastasis of carcinoma cells, is well documented and includes non-small lung, breast and prostate cancers [18,19,20,21], as well as melanoma [22], cervical carcinoma [23], and osteosarcoma [24]. In this respect, selective inhibition of UCH-L1 DUB activity with the available specific small-molecule inhibitors [25,26] might be valuable for the prevention of metastasis of cancer [3,27].

The membrane trafficking pathways in the transformed epithelial cells are central to the processes of invasion and metastasis effecting not only intercellular processes, but cell-cell communication as well [28,29,30,31,32,33]. Although UCH-L1 is mainly known as a deubiquitinating enzyme (DUB), its other activities have also been reported [34,35,36]. Endogenous UCH-L1 can be found in virtually any cell part and organelle including intra- and extra-cellular membrane structures. Our recently published work demonstrates that UCH-L1 membrane-anchoring function is required for targeting of the viral pro-metastatic molecule LMP1 to extracellular vesicles, exosomes; the processes of such sorting is mediated by C-terminal farnesylation of UCH-L1 [37]. In the present study we show that deubiquitinating activity of UCH-L1 is positively involved in UCH-L1-mediated membrane trafficking, and that specific abolishing of deubiquitinating function reduces the invasive potential of metastatic cells.

Recently published data demonstrate that inhibition of UCH-L1 DUB activity with the small molecule inhibitor LDN-57444 (which shows specific effects on UCH-L1 compared with other members of the UCH family [25] results in profound anti-metastatic effects in a mouse model of invasive carcinoma [38]).

Unfortunately, the limited aqueous solubility of LDN-57444 remains a challenge for further evaluations and clinical development. Therefore, we developed a nanoparticles formulation of LDN-57444, by incorporation of the compound in polyoxazoline micelles (LDN-POx). We have previously shown that nanoparticle-sized micelles formed from poly(2-oxazoline) amphiphilic block copolymers (POx co-polymer) can be used to deliver poorly soluble drugs and drug combinations [39,40,41]. The POx polymer micelle system is unique in its ability to incorporate unprecedentedly large amounts of insoluble drugs [42].

In this series of experiments, we show that inhibition of UCH-L1 DUB activity with LDN-57444 reduces invasive potential of malignant carcinoma cells. Based on our results, we propose that nanoparticles formulation of the LDN-57444 offers a useful additional approach to clinical development of anti-invasive therapy of metastatic carcinomas including EBV-associated cancers.

## 2. Results

**Figure 1:**
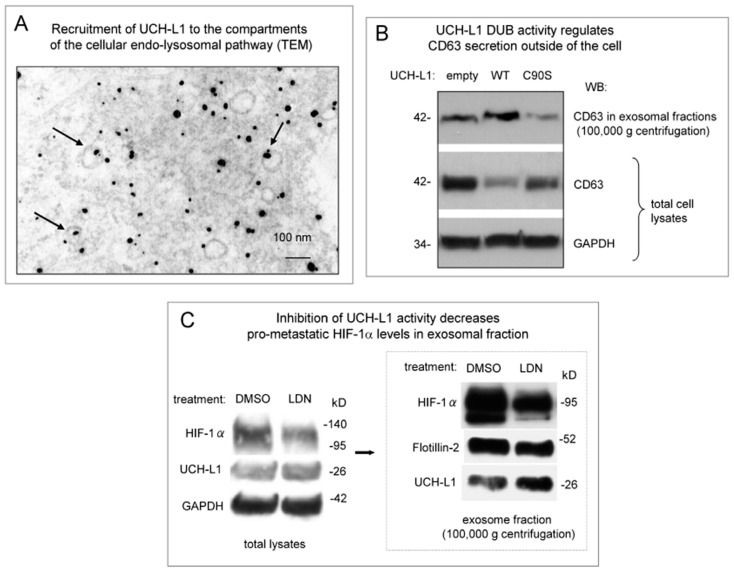
Ubiquitin carboxyl-terminal hydrolase L1 (UCH-L1) de-ubiquitinating activity is involved in regulation of membrane trafficking pathways. (**A**) UCH-L1 is physically associated with the vesicles of the cellular endolysosomal pathway. Electron microscopy of 293 cells immune gold-labeled for UCH-L1 (10 nm gold particles, black dots). Two hundred and ninety-three cells transfected with UCH-L1 expression vector were subjected to ultrastructural examination. Fixed ultra-sections were stained with UCH-L1 primary and colloidal gold-labeled secondary antibodies. Arrows show the intra-cellular compartments of the endo-lysosomal pathway. (**B**) UCH-L1 de-ubiquitinating (DUB) activity is involved in regulation of CD63 secretion in exosomes. Two hundred and ninety-three cells were transfected with WT and C90S UCH-L1 expression vectors. After 48 h of incubation, exosomes were purified by sequential ultracentrifugation as described in Materials and Methods section. Western blot analysis demonstrates that CD63 levels in the exosome fraction were reduced when the cells were transfected with DUB-dead UCH-L1 mutant compared to WT-expressing control. Western blot for CD63 in total lysates of the same cells confirms the results in exosomal fractions showing reverse correlation in CD63 levels between WT- and C90S-expressing cells (normalization to GAPDH). (**C**) Inhibition of UCH-L1 DUB activity with the selective small-molecule inhibitor LDN-57444 decreases the levels pro-metastaticHIF-1α in exosomal fractions. After transfection with hypoxia-inducible factor 1α (HIF-1α expression vector 293 cells were treated with three μM of LDN-57444 or DMSO as a control. After 48 h exosome fractions were purified by ultracentrifugation as described in Materials and Methods. The results of Western blot analysis of total cellular lysates and exosomal fractions show that HIF-1 α levels in the exosomal fraction were reduced when UCH-L1 DUB activity was inhibited. Protein levels in the exosomal fractions were normalized on UCH-L1 levels and on the exosome marker Flotillin-2. GAPDH served as normalization control for total lysates.

We have recently shown that C-terminal farnesylation of UCH-L1 is required for exosomal cargo loading [37]. At the same time, the results of our experiments indicated that de-ubiquitinating activity of UCH-L1 is also likely to be involved in exosome function as well [37]. Therefore, we first conducted tests to confirm the significance of endogenous UCH-L1 and its DUB activity for intra- and intercellular membrane trafficking (Figure 1). We used transmission electron microscopy (TEM) to examine whether endogenous UCH-L1 is associated with membrane structures inside 293 cells (which express relatively high levels of UCH-L1). As shown in Figure 1A, certain amounts of endogenous UCH-L1 are visibly attached to the membrane or present inside of the cytoplasmic membrane vesicles, presumably as components of the endo-lysosomal pathway. When expressed, UCH-L1 is associated with all major cellular systems involved in membrane trafficking, including extracellular membrane vesicles [23,43,44,45]. In our recent study we show that farnesylation of UCH-L1 and its de-ubiquitinating activity both play role in membrane trafficking of the transformed cells [37]. We have now examined whether UCH-L1 DUB activity specifically affects exosome release. As shown in Figure 1B, over-expression of WT UCH-L1 increase secretion of CD63 outside the cells, while enzymatically inactive mutant UCH-L1 C90S reduced CD63 in exosomal fractions obtained from cell supernatant fluid. Since CD63 is commonly accepted as one of the exosomal markers, this result is indirect evidence that UCH-L1 DUB activity is involved at least in final steps of exosome biogenesis and secretion.

Recent studies show that UCH-L1 is involved in cancer progression, invasion and metastasis [3,8]. One of the well-established pro-metastatic factors, hypoxia-inducible factor 1α (HIF-1α) is known to be induced by the viral oncogene LMP1 in EBV-positive metastatic nasopharyngeal cancer (NPC) cells [46,47,48]. In addition, we have shown that HIF-1α can be transferred with nasopharyngeal carcinoma-associated LMP1-positive exosomes, and that such transcriptionally active HIF-1α supports the invasive potential of NPC [38]. We now test whether UCH-L1 DUB activity participates in targeting HIF-1α to exosomes in LMP1-expressing cells. In this experiment we have used a well-documented inhibitor of UCH-L1 DUB activity, LDN-57444 [25,38,49,50]. The results normalized to GAPDH, show that treatment of cells with 3 μM of LDN-57444 for 48 h resulted in some reduction of HIF-1α levels in total lysates (Figure 1C, left). The effects likely mediated via HIF-1α more intense proteasomal degradation in the presence of UCH-L1 inhibitor [38]. However, the effect of LDN-57444 was much more profound in the exosomal fractions from the treated cells (Figure 1C, right): The results after normalization on the exosomal marker flottilin-2 and UCH-L1 levels, demonstrate significantly less amounts of HIF-1α in exosomes after LDN treatment. Taken together, these results indicate that UCH-L1 de-ubiquitinating activity (along with its farnesylation capacity [37]) is required for membrane trafficking in transformed cells, including through exosome-mediated transfer of pro-metastatic molecules. Therefore inhibition of UCH-L1 DUB function with LDN-57444 might be as well beneficial for EBV-positive metastatic cancers, as it was in a murine model of pulmonary metastasis [38].

LDN-57444 is a promising small molecule inhibitor of UCH-L1 DUB activity, it is specific for UCH-L1 compared to other UCHs at <10 μM [25]. However, its very low water solubility presents a common challenge to successful drug development and requires a proper formulation design to achieve therapeutic outcome [51].

**Figure 2:**
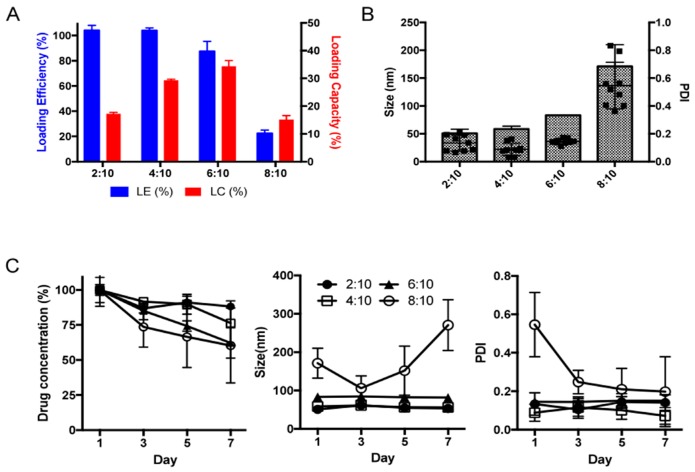
UCH-L1 inhibitor LDN-57444 can be effectively loaded into polymeric micellar system based on amphiphilic polyoxazoline block copolymer. (**A**) Loading efficiency (LE) and loading capacity (LC) of LDN-POx micelles at LDN:POx *w*/*w* ratios of 2:10, 4:10, 6:10 and 8:10. LE of LDN-POx micelles remained close to 100% up to 6:10 LDN:POx ratio, resulting in LC of 35% *w*/*w*. (**B**) Particles size and particles size distribution of LDN-POx micelles at LDN:POx *w*/*w* ratios of 2:10, 4:10, 6:10 and 8:10. LDN-POx micelles formed small (50–70 nm), homogenous particles (polydispersity index (PDI) approx. 0.2) up to 6:10 LDN:POx ratio. (**C**) Stability of LDN-POx micelles stored at 4 °C overtime as determined by changes in drug loading, particles size and particles size distribution. LDN-POx micelles prepared at LDN:POx ratio of up to 6:10 were stable during incubation for up to 7 days.

To overcome the problem of the potential drug’s bioavailability, we have incorporated the drug in polymeric micellar system based on amphiphilic block copolymer, poly(2-methyl-2-oxazoline)-poly(2-butyl-2-oxazoline)-poly(2-methyl-2-oxazoline) (PMeOx-PBuOx-PMeOx). We generated micelles with LDN-57444:POx ratios of 2:10, 4:10, 6:10, 8:10 *w*/*w* (drug to polymer) using the previously described thin film method. The highest drug loading capacity in POx micelles was achieved at the drug:polymer ratio of 6:10 and the loading capacity was about 35% (Figure 2A). The particles size of LDN-POx micelles was below 100nm and the polydispersity index (PDI) of 0.2 indicated homogeneous particles size distribution (Figure 2B). The formulations were stable in aqueous solution for 7 days, without changes in the particles size or signs of drug precipitation, although slight decrease in drug loading was observed for 6:10 drug:polymer LDN-POx micelles (Figure 2C). However, the further increase in the drug:polymer ratio didn’t improve the loading capacity. Particles size and size distribution increased as well (0.6) and drug precipitation was observed in stability studies. Taken together, we used the LDN-POx 2:10 formulation to the in-vitro efficacy studies.

**Figure 3:**
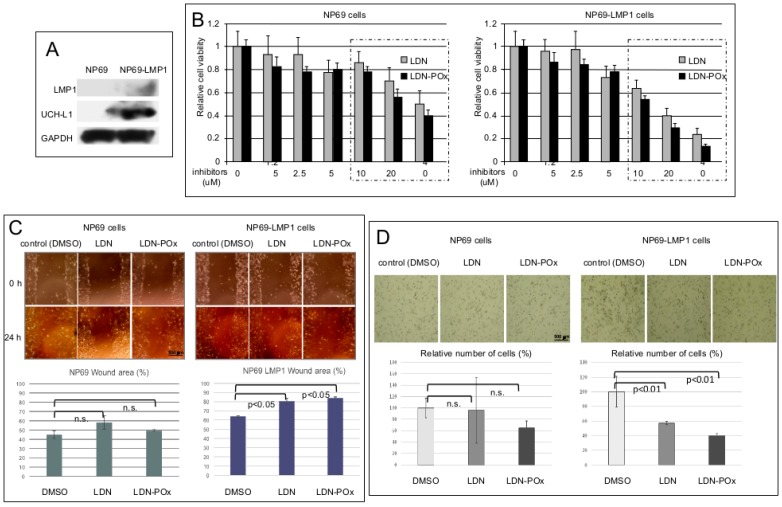
Both forms of UCH-L1 inhibitor, LDN and LDN-POx reduce motility of LMP1-positive nasopharyngeal cells. (**A**) De novo expression of UCH-L1 in LMP1-positive NP cells. Total lysates from LMP1-negative and LMP1-positive cells NP69 cells were separated in 12% polyacrylamide gel electrophoresis (PAGE), and expression of endogenous UCH-L1 protein was determined in by Western blot analysis as described in Materials and Methods. GAPDH levels served as loading control. (**B**) Determination of non-toxic concentrations of LDN and LDN-POx forms of the UCH-L1 inhibitor. NP69 and NP69-LMP1 cells were seeded in culture plates at a density of 2–5 × 10^3^ cells/well and either DMSO (control) or indicated concentrations of free LDN-57444, or micellated LDN-POx forms of the LDN-57444 inhibitor were added to each well. 72 h later 3-(4,5-dimethylthiazol-2-yl)-5-(3-carboxymethoxyphenyl)-2-(4-sulfophenyl)-2H-tetrazolium salt (MTS) assays were performed as described in Materials and Methods. The results demonstrate that concentrations of the inhibitor less than 5 μM did not affect the viability of either cell line. (**C**) Inhibition of UCH-L1 DUB activity reduces motility of only LMP1-positive NP69 cells, but not those that are LMP1-negative. Sub-confluent LMP1-negative and -positive NP69 cells had been incubated with or without 3 μM LDN or LDN-POx for 48 h. Then the cells were scratched and cultured for additional 24 h. The widths of the ‘wound’ (scratched areas) were measured by ImageJ software (http://rsb.info.nih.gov/ij/) and the percentage of the wound healed was calculated by the following formula: ‘Wounded area filled (%)’ = 100% (width after 24 h/width at beginning) × 100% as shown in the histogram. On the top are representative images of control, LDN and LDN-POx-treated NP69 and NP69-LMP1 cells at 0 h and 24 h after the scratch was applied (* *p* < 0.01, *T*-test). (**D**) Inhibition of UCH-L1 DUB activity reduces invasive capacity of only NP69 cells expressing LMP1/UCH-L1. LMP1-negative and LMP1-positive NP69 cells treated with 3 μM of LDN and LDN-POx were added to the insert of a transwell cell-culture chamber containing Matrigel and incubated for 24 h. Medium containing 10% fetal bovine serum was added to the bottom of the chamber. Cells that attached to the underside of the membrane were fixed, stained with hematoxylin and counted, and the average number of cells per field of view was determined (mean ± s.d.; *n* = 3). Scale bar (**C** and **D**): 500 μm.

Initially, we tested two forms of LDN inhibitor of UCH-L1 DUB activity on the viability and migration of the well-established nasopharyngeal NP69 parental control line, and on the NP69 cell line stably expressing EBV pro-metastatic factor, LMP1 (Figure 3). Since LMP1 induces metastatic changes in NP69 cells [52,53,54,55], and UCH-L1 is expressed de novo in rather metastatic than in primary carcinomas [3,56], we first confirmed our previous observation that LMP1 induces expression of UCH-L1 in adherent cells [13]. Figure 3A demonstrates a visible boost in UCH-L1 expression levels in LMP1 stably-expressing NP69 cell line. Next, we determined the concentrations of both “free” (LDN) and “micellated” (LDN-Pox) forms of LDN-57444 that would not affect cell viability. The results of 3-(4,5-dimethylthiazol-2-yl)-5-(3-carboxymethoxyphenyl)-2-(4-sulfophenyl)-2H-tetrazolium salt (MTS) assay in Figure 3B show that in concentration higher than 5 μM both forms of inhibitor start inducing death in both NP69 and NP69-LMP1 cells definitely through non-specific, UCH-L1-independent pathways. Based on this data, we have decided to use the concentration of LDN and LDN-POx at 3 μM for specific inhibition of UCH-L1 DUB activity in our cell culture experiments.

We next examined whether inhibition of UCH-L1-dependent de-ubiquitination would have physiological effects on the motility (and therefore invasive potential) of NP69 and NP69-LMP1 cells (Figure 3C,D). Since only LMP1-positive cells express UCH-L1, specific inhibition of UCH-L1 DUB activity should affect only NP69-LMP1 cell migration. We performed the wound-healing and Matrigel invasion assays in both cell lines: Sub-confluent cells were treated with dimethyl sulfoxide (DMSO) (control) and LDN and LDN-Pox forms of UCH-L1 inhibitor, scratched, and incubated for 24 h with 3 μM of LDN-57444 in both forms (Figure 3C). For Matrigel invasion assay the suspension of both cell lines treated with each drug was added to the insert of a transwell cell-culture chamber containing Matrigel. After 24 h attached cells were fixed and counted (Figure 3D). The results show that UCH-L1 inhibitors (LDN and LDN-POx) significantly suppressed only migration of LMP1-positive cells (expressing UCH-L1), but not the motility of the control NP69 cell line. The distances between the wound edges were measured; the graphs in Figure 3C, bottom panels, show that the differences in cell migration between control NP69 cell line and NP69-LMP1 UCH-L1-expressing cells were statistically significant.

**Figure 4:**
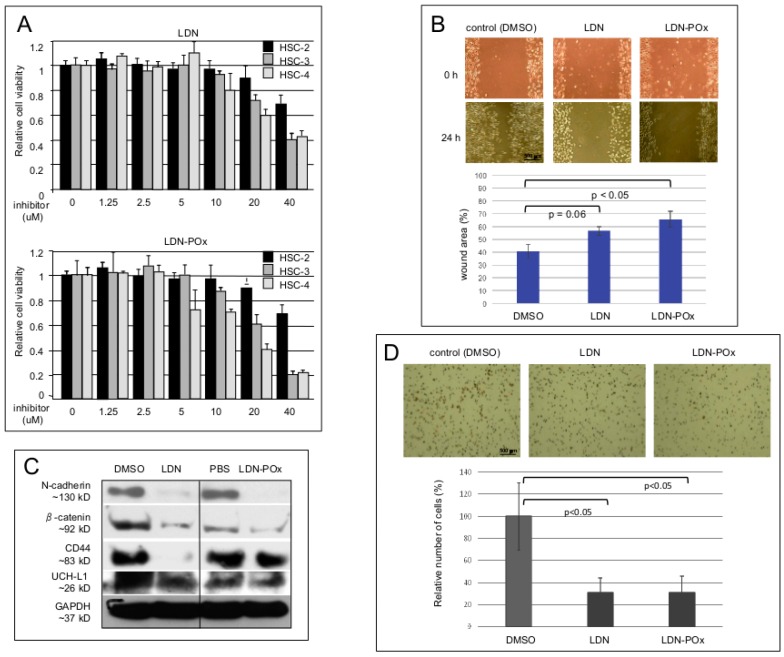
Both LDN and LDN-POx decrease expression of pro-metastatic factors as well as motility of oral squamous carcinoma (OSC) cells. (**A**) Determination of non-toxic concentrations of LDN and LDN-POx forms of the UCH-L1 inhibitor in metastatic human squamous carcinoma cells (HSC). HSC-2, -3 and -4 cells were treated with LDN or micellated LDN-POx forms of the UCH-L1 inhibitor and MTS assays were performed as described in Figure 3B. The results demonstrate that concentrations of the inhibitor less than 5 μM did not affect the viability of all three cell lines. (**B**) Inhibition of UCH-L1 DUB activity reduces motility of highly metastatic HSC-3 cells. The cells were treated with either LDN or LDN-POx (3 μM each) (DMSO as control). After 48 h incubation a scratch assay was performed as described in Figure 3C. The results of statistical analysis on the bottom indicate that both LDN and LDN-POx had inhibitory effect on HSC-3 cells motility. (**C**) Inhibition of UCH-L1 DUB activity reduces the expression of pro-metastatic factors in HSC-3 cells. Total lysates from HSC-3 cells treated for 72 h with both, free and micellated forms of UCH-L1 inhibitor (DMSO and PBS served as control respectively) were separated in 4–20 gradient PAGE, and Western blots with indicated antibodies (N-cadherin, β-catenin and CD44) were performed. Protein levels were normalized on GAPDH and UCH-L1. (**D**) Inhibition of UCH-L1 DUB activity reduces invasive capacity of HSC-3 cells. The cells treated with 3 μM of LDN and LDN-POx (DMSO-treated cells are shown as control) were added to the insert of a transwell cell-culture chamber containing Matrigel and incubated for 24 h. Medium containing 10% fetal bovine serum was added to the bottom of the chamber. Cells that attached to the underside of the membrane were fixed, stained with hematoxylin and counted, and the average number of cells per field of view was determined (mean ± s.d.; *n* = 3). Scale bar (**B** and **D**): 500 μm.

Next, we investigated the physiological effects of UCH-L1 DUB activity inhibition in several epithelial cell lines established from metastatic human oral squamous carcinomas (HSC cell lines). The goal of these experiments was to determine what effects inhibition of UCH-L1 enzymatic activity would have on the cell lines in terms of different levels of invasiveness [57,58]. As in the experiments with NP-69 cells (Figure 3A), we first established the non-toxic concentrations of both forms of LDN-57444 inhibitor in MTS assay (Figure 4A). Figure 4B demonstrates that non-toxic concentration of both, LDN and LDN-POx forms of the inhibitor significantly reduce motility of the most invasive, HSC-3 [58] cells as wound-healing assay shows. It is worth noting that similar effects were observed in other HSC cells, and for LDN-POx we also used phosphate-buffered saline (PBS) control.

Since the increased motility and invasiveness of metastatic carcinomas directly correlates with the expression of certain pro-metastatic adhesion molecules [59], we next examined whether inhibition of UCH-L1 DUB activity will down-regulate established metastatic markers such as N-cadherin, β-catenin and CD44. The results of the western blot analysis in Figure 4C demonstrate that treatment of highly metastatic HSC-3 cells with 5 μM of both forms of LDN-57444 inhibitor for 24 h significantly decreased expression of the endogenouastatic markers compared with the control cells (although the inhibitory effect of LDN-POx on CD44 was less profound than the reduction of CD44 protein levels under non-micellated form of the inhibitor). In addition, to confirm anti-metastatic effects of LDN and LDN-POx forms of UCH-L1 DUB inhibitor, we performed a Matrigel invasion assay with HSC-3 cells (Figure 4D).

**Figure 5:**
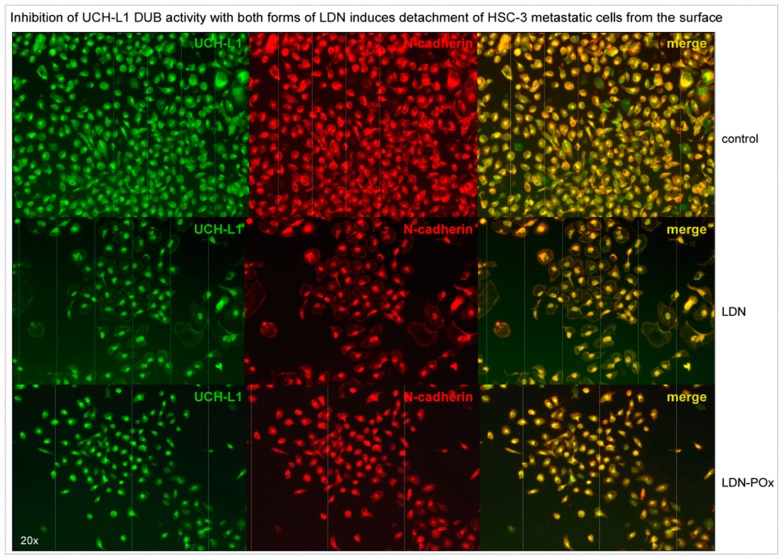
Inhibition of UCH-L1 DUB activity with LDN or LDN-POx induces detachment of metastatic carcinoma cells from the surface. Equal amounts of HSC-3 cells were plated at ~70–80% confluence and treated with free LDN and LDN-POx micellated forms of UCH-L1 inhibitor (3 μM each, DMSO as control). For 24 h the cells were washed with phosphate-buffered saline (PBS), fixed in 4% Paraformaldehyde (PFA) and fluorescent immunostaining was performed with UCH-L1 and N-cadherin (as a marker of metastatic carcinoma cells) antibodies. Scale bar: 200 μm.

Considering that inhibition of UCH-L1 DUB activity reduced expression of membrane receptors involved in cellular adhesion (Figure 4C), we decided to evaluate how prolonged incubation with both forms of LDN-57444 inhibitor would affect adhesion and morphology of highly metastatic HSC-3 cells. The same amounts of the cells were plated on the coverslips and treated with 3 μM of both forms of LDN inhibitor. As immunostaining analysis in Figure 5 demonstrates that after 72 h of treatment, the cultured cells were detached from the surface with approximately 30–50% cells left. The floating cells were collected and analyzed for viability by MTS assay. At 72 h time point approximately 40–60% of the floating cells were still alive, but after 96 h the detached cells were dead. Together, the results of the experiments in Figure 4 and Figure 5 provide evidence that the use of small molecule inhibitors of UCH-L1 DUB activity might be beneficial for treatment of selected metastatic carcinomas.

**Figure 6:**
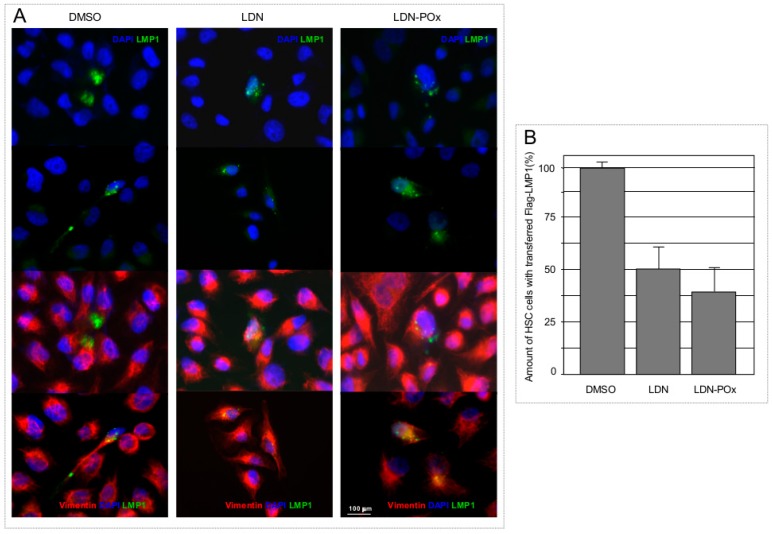
Both, free and micellated forms of UCH-L1 DUB inhibitor prevent extra cellular vesicles (ECVs)-mediated transfer of viral oncogene LMP1 to carcinoma cells. (**A**) Two hundred and ninety-three cells were transfected with Flag-LMP1-expressing (vector) and 3 μM of LDN or LDN-POx UCH-L1 inhibitors were added to the cells for 24 h. Extracellular vesicles (ECVs) from transfected and treated 293 cells were purified, as described in Materials and Methods. Growing HSC-3 cells were washed tree times with PBS and treated with ECV in fresh media with 10% fetal bovine serum (FBS) at ~70% confluence. Twenty-four hours later the cells were fixed in 4% PFA and co-stained with Flag and vimentin antibodies. Shown are representative images (4′,6-diamidino-2-phenylindole (DAPI) staining shows nuclei). (**B**) LMP1-positive cells were counted in three random fields, and the results are presented as a percentage of HSC-3 cells with Flag-LMP1 transferred with ECVs. Scale bar: 100 μm.

Since UCH-L1 is a multifunctional molecule [6], and is known to participate in formation and function of the components of the cellular cytoskeleton in transformed cells [60], it is expected that different cellular functions of UCH-L1 will be involved in basic processes such as cellular membrane trafficking. In our recently published work we have demonstrated that although farnesylation of UCH-L1 is definitely significant for targeting of LMP1 (and likely some other membrane-associated cargo molecules) to exosomes [37], blocking UCH-L1 DUB activity with the expression of UCH-L1 DUB-dead mutant somewhat reduces cargo loading to the exosomes as well ([37] and Figure 1). Moreover, in our previously published work we have shown that the EBV oncogene LMP1, along with other pro-metastatic molecules such as HIF-1α co-transferred with exosomes, promotes invasiveness in recipient cells by inducing epithelial-mesenchymal transition (EMT)-associated markers [61]. Therefore, in the next experiment, we examined whether transfer of LMP1-positive extra cellular vesicles (ECVs) can be reduced by inhibition of UCH-L1 de-ubiquitinating activity in donor cells. Results in Figure 6 demonstrate that treatment of ECVs from LMP-transfected cells with both LDN and LDN-POx inhibitors resulted in reduced ECV-mediated transfer of LMP1 to the LMP1-negative carcinoma cells (Figure 6B). This result indicates that ECV-mediated transfer of pro-metastatic molecules at least partially depends on the de-ubiquitinating activity of UCH-L1 presents in exosomes (Figure 1C and [37]) and other extracellular vesicles [37].

## 3. Discussion

Closely involved in the regulation of numerous signaling pathways in tumor cells, the ubiquitin system has become an attractive target for anti-cancer therapy in the last several years. However, most such drugs inhibit proteasome function, while the physiological role of ubiquitination and de-ubiquitination is much broader than just marking proteins for proteasomal degradation. Targeting specific ubiquitinating and de-ubiquitinating enzymes of the ubiquitin-dependent regulatory network might produce more selective effects.

The physiological roles of UCH-L1 and regulation of its expression and activities in normal and transformed cells remains largely unexplored, however, recent information on de novo expression and functional activity of UCH-L1 indicates its involvement in critical cellular processes involved in oncogenesis of human B-lymphomas [7,12,15,62,63]. In the case of carcinomas, it seems that UCH-L1 expression has been observed rather in metastatic cells than in primary carcinomas [56,64,65,66,67]. UCH-L1 induced the expression of metastasis-related genes and promotes distant tumor metastases [38], which demonstrates a potential role for this multifunctional molecule in cancer progression and development. This expands the specific biological role of UCH-L1 dramatically: UCH-L1 might represent a novel molecular target for the design of inhibitors with anti-metastatic potential [3,38].

Tumor-microenvironment interactions are increasingly recognized to influence tumor progression. In head-and-neck squamous cell carcinoma, including NPC, secretion of extra-cellular vesicles (ECVs) is associated with advanced stages of the disease. In most cases, those vesicles such as exosomes, ectosomes and apoptotic bodies have been shown to mediate progression, metastasis, survival, drug resistance, immune modulation, and many other aggressive cancer phenotypes [68,69,70,71,72,73,74,75].

Recently published data by several groups including ours [37,76], and the results present in Figure 1 clearly show the involvement of UCH-L1 in regulation of membrane trafficking pathways including formation and function of ECVs. Of particular note is that separate functional characteristics of UCH-L1, such as farnesylation [37] and de-ubiquitinating activity (Figure 1B,C), are both important for the proper function of ECV-associated cellular system.

The possibility of boosting UCH-L1 de novo expression in nasopharyngeal cells by the EBV oncogene LMP1 (Figure 3A), a well-established viral pro-metastatic factor in nasopharyngeal carcinomas [77,78,79], provided an ideal cellular test system for the UCH-L1 selective inhibitor LDN-57444: Both, “free” and “micellated” forms of the compound statistically reduced migration of only LMP1/UCH-L1-positive cells but not the paternal, non-invasive NP69 cells that do not express any significant levels of UCH-L1 protein (Figure 3A,C,D). These results affirm that non-toxic concentrations of LDN and LDN-POx might be selectively effective against only UCH-L1-positive invasive carcinoma cells. In previously published work on inhibition of UCH-L1 DUB activity, in vivo Goto Y. and co-authors note that they did not observe any obvious side effects after the administration of LDN-57444 indicating that UCH-L1 is a good therapeutic target for the suppression of distant tumor metastases [38].

Interestingly, in our cell culture wound closure assays the soluble form (LDN-POx) of the UCH-L1 inhibitor was as effective as the original one (LDN) (Figure 3C and Figure 4B). Nevertheless, the Matrigel invasion assays demonstrated that the cell invasion activity of LMP1-expressing nasopharyngeal cells is significantly more inhibited by the soluble form of LDN-57444 (Figure 3D), but not the invasiveness of the squamous carcinoma cells (Figure 4D). Future investigation will determine whether the nanoparticle formulation of LDN-57444 is more effective in EBV-positive metastatic carcinomas.

Development of nanosized, stable formulation of LDN-57444 in POx micelles opens the pathway to drug evaluations in vivo and clinical translation. Furthermore, the flexibility and richness of the polymer chemistry allows for the development of formulations of additional pathway inhibitors alone or in combination with other synergistic therapeutic agents.

Since there is evidence that high levels of UCH-L1 expression correlate with the more progressive, metastatic stages of malignancies, the inhibitory effects of LDN-POx on UCH-L1 DUB activity would be beneficial specifically for poorly differentiated and highly invasive carcinomas such as oral squamous cell carcinoma (OSCC) and EBV-positive NPC.

## 4. Materials and Methods

### 4.1. Preparation and Characterization of POx/LDN-5744 Micelle

The amphiphilic triblock copolymer [P(MeOx_37_-*b*-PBuOx_21_-*b*-PMeOx_36_)), Mn = 8.25 kg/mol, PDI (DM = 1.21)] was synthesized as reported previously by using living cationic ring-opening polymerization and used to prepare LDN-57444 loaded polymeric micelle formulation (LDN-POx) via the thin film hydration method [39]. Briefly, predetermined amounts of polymer and LDN-57444 were each dissolved in organic solvents and mixed together. The organic solvent was then evaporated under a stream of nitrogen gas (50 °C) to form the thin film. To completely remove the residual organic solvent, the films were deposited in the vacuum chamber (approx. 0.2 mbar) overnight. Subsequently, the thin films were rehydrated with saline and then incubated at room temperature for 10 min to produce LDN-57444 loaded polymeric micelle formulations. The aqueous polymeric micelle formulation was centrifuged at 10,000 rpm for 3 min (Sorvall Legend Micro 21R Centrifuge, Thermo Scientific) to precipitate non-dissolved drug or drug-polymer aggregates. The transparent supernatant solutions of micelle samples were used for the further analysis. The hydrodynamic diameter and polydispersity index (PDI) of LDN-57444 loaded polymeric micelles were determined by dynamic light scattering (DLS) using Malvern Nanosizer.

### 4.2. High-Performance Liquid Chromatography (HPLC) Analysis of LDN-57444 in POx Micelle

The quantitative analysis of LDN-57444 in polymeric micelles was performed by an HPLC system (Agilent Technologies 1200 series) using Agilent eclipse plus C18 3.5 μm column (4.6 mm × 150 mm). The micelle samples were diluted with mobile phase (a mixture of acetonitrile/water (30%/70% v/v, 0.01% trifluoroacetic acid) and injected (10 μL) into the HPLC system. The flow rate was 1.0 mL/min, and column temperature was 40 °C. The detection wavelength was 245 nm.

The equations as follow were used to calculate loading efficiency (LE) and loading capacity (LC):

$$LE\;\left(\%\right)=\frac{m_{drug}}{m_{drug_{\;}added}}\times 100$$

$$LC\;\left(\%\right)=\frac{m_{drug}}{m_{drug}+m_{excipient}}\times 100$$

where *m_drug_* and *m_excipient_* are the mass of the solubilized drug and polymer excipient in the solution, while *m_drug added_* is the weight amount of the drug added to the dispersion. Drug concentration (DC) was determined by HPLC and calculated against free LDN-57444 standards.

### 4.3. Stability Study of POx/LDN-57444 Micelle

Stability study of LDN-POx micellar formulations were performed via monitoring both the drug content in polymeric micelle (to confirm the stability of the encapsulated LDN-57444 in micelle formulation) and the size and size distribution of the LDN-POx micelles. The LDN-POx micelles were incubated in saline at polymer concentration of 10 mg/mL at 4 °C. The solutions were sampled every other day up to day 7 and the LDN-5744 content in POx micelles was determined by HPLC and size and size distribution were measured by DLS as described above.

### 4.4. Cell Culture

Two hundred and ninety-three cells, human embryonic kidney cells, NP69 and NP69LMP1, human immortalized NP epithelial cells, and HSC-2, HSC-3, and HSC-4, oral carcinoma cell lines, were used for these experiments. NP69 and NP69LMP1, human immortalized NP epithelial cells were kindly provided by Dr. Sai Wai Tsao (University of Hong Kong, Hong Kong, China). All cells were maintained in Dulbecco’s modified Eagle medium (DMEM) at 37 °C in 5% CO_2_. On assays related to exosomes, fetal bovine serum (FBS) was depleted of bovine exosomes by ultracentrifugation at 100,000× *g* for 60 min.

### 4.5. Chemical Agents

LDN-57444, 2-methyl-2-oxazoline, and other reagents for synthesizing 2-butyl-2-oxazoline (valeronitrile, cadmium acetate dehydrate and ethanolamine) and polymer (acetonitrile, n-boc-piperazine) were obtained from Sigma-Aldrich Inc (St. Louis, MO, USA).

#### Antibodies

Antibodies were purchased as indicated: UCH-L1 (381000) from Thermo Fisher Scientific, Rockford, IL USA; HIF-1α (sc-10790), N-cadherin (sc-271386), and Vimentin (sc-66002) from Santa Cruz Biotechnology, Santa Cruz, CA, USA; Flotillin-2 (610383) from BD Biosciences, San Jose, CA, USA; Flag (F3165), CD44 (SAB4300691), and CD63 (SAB4301607) from Sigma-Aldrich, St. Louis, MO, USA; GAPDH (H00002597-M3) from Abnova, Taipei, Taiwan; anti-mouse (NA931V) and anti-rabbit (NA934V) secondary antibody for western blotting was purchased from GE Healthcare, Little Chalfont, UK; secondary antibody for immunoflorescence microscopy, donkey anti-mouse Alexa Flour 488 (A-21202) and 594 (A-21203) and donkey anti-rabbit Alexa Flour 488 (A-21206) and 594 (A-21207), was purchased from Thermo Fisher Scientific, Rockford, IL USA.

### 4.6. Immunoelectron Microscopy

Exosome-containing pellets obtained by ultracentrifugation were fixed with 4% paraformaldehyde in PBS for 2 h at room temperature and then resuspended in 2% paraformaldehyde in PBS. Droplets of this exosomal fraction were directly spotted onto Formvar/carbon-coated copper grids (100 meshes) and incubated with anti-UCH-L1 antibody. After several rinses in PBS-0.1% bovine serum albumin, the grids were incubated with 18-nm diameter colloidal gold particles (prepared by citrate method) conjugated with protein A (Pharmacia, Uppsala, Sweden) and diluted in 1% bovine serum albumin in PBS. Control experiments were performed by omission of the primary antibodies from the labeling procedure. Finally, grids were stained with a solution of 2% methyl cellulose and 0.4% uranyl acetate before examination by transmission electron microscopy.

### 4.7. Immunoflorescence Microscopy

Cells cultured on glass coverslips were fixed with 4% paraformaldehyde, washed with phosphate-buffered saline (PBS), permeabilized with 1% Triton X-100/PBS and blocked with 2% normal donkey serum. Cells were incubated with primary antibodies overnight at 4 °C and then incubated with secondary antibodies for 1 h at room temperature.

### 4.8. Western Blotting Analysis

Total cell lysates were denatured in sodium dodecyl sulfate (SDS) (Sigma, St. Louis, MO, USA) loading buffer and boiled for 5 min. Samples were separated by SDS-polyacrylamide gel electrophoresis (SDS-PAGE) and transferred to polyvinylidene fluoride (PVDF) membranes (Bio-Rad Laboratories, Hercules, CA, USA). Membranes were blocked with 5% milk in Tris-buffered saline-Tween 20 (TBST) and incubated overnight at 4 °C with primary antibodies. Membranes were then washed and incubated with appropriate horseradish peroxidase-conjugated secondary antibodies for 1 h at room temperature. Membranes were washed again, and bands were visualized with enhanced chemiluminescence reagent (Advansta, Menlo Park, CA, USA).

### 4.9. Transient Transfection

Two hundred and ninety-three cells were grown in 100 mm plates and transfected with 3 μg of plasmids with the use of polyethylenimine (VWR, Radnor, PA, USA). An empty vector was used to equalize total amounts of DNA in the transfections.

### 4.10. Matrigel Invasion Assay

The suspension of NP69 and HSC-3 cells incubated with each drug was added to the insert of a transwell cell-culture chamber containing Matrigel and incubated for 24 h. Medium containing 10% fetal bovine serum was added to the bottom of the chamber. Cells that attached to the underside of the membrane were fixed, stained with Hematoxylin and counted, and the average number of cells per field of view was determined (mean ± s.d.; *n* = 3; Dunnett-test).

### 4.11. Exosome Isolation

Exosomes were purified by sequential centrifugation as previously described [61]. In brief, indicated cells were grown with 10% exosome-free FBS containing DMEM, then cell culture supernatant was collected and removed any cell contamination by centrifugation at 400× *g* for 5 min. To remove large cell debris, the supernatants were then spun at 2000× *g* for 10 min. Then ectosome fraction was collected by centrifugation at 20,000× *g* for 60 min. Finally, exosome fraction was collected by centrifugation at 100,000× *g* for 60 min. Exosomes and ectosomes were washed in PBS and pelleted again by centrifugation at the same speed.

### 4.12. Extracellular Vesicles Isolation and Transfer

A total of 293T cells were grown to confluent monolayers and grown in culture for an additional day. ECV were collected by differential centrifugation from conditioned media, resuspended into PBS, and stored at −80 °C until use [37]. Cell monolayers were washed twice with PBS and incubated with purified exosomes for the indicated times at 37 °C in serum-free media. Cells exposed to exosomes were washed three times with PBS, scraped into cold PBS, pelleted, and lysed in PBS. HRE-luciferase plasmid and β-galactosidase plasmid were transfected into 293T cells before exposure to exosomes.

### 4.13. Wound-Healing Assays

For wound-healing assays, confluent cell monolayers incubated with either free LDN-57444, LDN-micelle, or DMSO control were scratched with a micropipette tip, and spontaneous cell migration was monitored for 24 h. The widths of the ‘wound’ (scratched areas) were measured by ImageJ (http://rsbweb.nih.gov/ij/), and the proportion of wound was calculated by the following formula: (Width after 24 h/width at the beginning) × 100%.

### 4.14. In vitro Growth Inhibition Assay

A 3-(4,5-dimethylthiazol-2-yl)-5-(3-carboxymethoxyphenyl)-2-(4-sulfophenyl)-2H-tetrazolium salt (MTS) assay was performed to assess the effect of the UCH-L1 inhibitors on cell proliferation using the CellTiter 96 AQueous One Solution Cell Proliferation Assay (Promega Corp, Fitchburg, WI, USA) as described previously [80,81]. In brief, cells were seeded in 96-well culture plates at a density of 2–5 × 10^3^ cells/well, depending on cell lines, and either DMSO (control), a graded concentration of free LDN-57444, or LDN-micelle was added to each well. After either 48-h or 72-h incubation per with drugs, the MTS reagents were added to each well and incubated for 2 h. Optical density was read with Synergy 2 Multi-Mode Reader (BioTek, Winooski, VT, USA) at a wavelength of 490 nm.

### 4.15. Statistical Analysis

Error bars in graphical data represent mean ± SD unless otherwise specified. Statistical significance was analyzed using Welch *t*-test. On analysis between more than 3 samples, Dunnett’s test was used to avoid multiple comparisons problem. All statistical analysis was performed using EZR software [82]. A value of *p* < 0.05 was considered to be significant.
